# Viral and Bacterial Pathogens in Bovine Respiratory Disease in Finland

**DOI:** 10.1186/1751-0147-45-193

**Published:** 2004-12-01

**Authors:** H Härtel, S Nikunen, E Neuvonen, R Tanskanen, S-L Kivelä, P Aho, T Soveri, H Saloniemi

**Affiliations:** 1 Department of Clinical Veterinary Sciences, University of Helsinki, Helsinki, Finland; 2 Järvi-Suomen Portti, Helsinki, Finland; 3 Department of Virology and Epidemiology, Virology Unit, Helsinki, Finland; 4 Department of Basic Veterinary Sciences, Faculty of Veterinary Medicine, University of Helsinki, Helsinki, Finland; 5 Department of Bacteriology, National Veterinary and Food Research Institute, Helsinki, Finland; 6 Kuopio Regional Laboratory, National Veterinary and Food Research Institute, Helsinki, Finland

**Keywords:** bovine respiratory disease, calf, pneumonia, bacteria, virus, mycoplasma, tracheobronchial lavage, seroconversion

## Abstract

Pathogens causing bovine respiratory tract disease in Finland were investigated. Eighteen cattle herds with bovine respiratory disease were included. Five diseased calves from each farm were chosen for closer examination and tracheobronchial lavage. Blood samples were taken from the calves at the time of the investigation and from 86 calves 3–4 weeks later. In addition, 6–10 blood samples from animals of different ages were collected from each herd, resulting in 169 samples. Serum samples were tested for antibodies to bovine parainfluenza virus-3 (PIV-3), bovine respiratory syncytial virus (BRSV), bovine coronavirus (BCV), bovine adenovirus-3 (BAV-3) and bovine adenovirus-7 (BAV-7). About one third of the samples were also tested for antibodies to bovine virus diarrhoea virus (BVDV) with negative results. Bacteria were cultured from lavage fluid and in vitro susceptibility to selected antimicrobials was tested. According to serological findings, PIV-3, BAV-7, BAV-3, BCV and BRSV are common pathogens in Finnish cattle with respiratory problems. A titre rise especially for BAV-7 and BAV-3, the dual growth of *Mycoplasma dispar* and *Pasteurella multocida*, were typical findings in diseased calves. *Pasteurella* sp. strains showed no resistance to tested antimicrobials. *Mycoplasma bovis* and *Mannheimia haemolytica* were not found.

## Sammanfattning

*Förekomst av luftvägspatogener vid enzootisk pneumoni i finska nötbesättningar*

Studien genomfördes under åren 1998–1999 för att få grundkunskap om förekomsten av infektionsämnenassocierade med kalvarnas luftvägsinfektioner i Finland. Alla 18 besättningarna hade problem med luftvägssjukdomar. I varje besättning utvaldes 5 sjuka kalvar för noggrann undersökning och tracheobronkiell sköljning. För serologiska undersökningar av virusantikroppar togs blodprov från 86 kalvar med 3–4 veckors mellanrum. Från samma besättningar togs dessutom 6–10 serumprov från nöt i olika åldersgrupper, sammanlagt 169 prov. Serumproverna analyserades för antikroppar mot parainfluensavirus typ3 (PIV-3), bovint respiratoriskt syncytialt virus (BRSV), bovint coronavirus (BCV), bovint adenovirustyp 3 (BAV-3) och bovint adenovirus typ 7 (BAV-7). En tredjedel av proverna analyserades dessutompå antikroppar mot bovint virusdiarrevirus (BVDV) med negativt resultat. De tracheobronkiella skjölproverna undersöktes på mykoplasmer och andra bakterier. Enligt serologiska resultat tycks PIV-3, BAV-7, BAV-3, BCV och BRSV förekomma allmänt hos besättningar med luftvägssjukdomar i Finland. Serokonversion konstaterades oftast mot BAV-7 och BAV-3. I bakteriologiska undersökningar av sköljprovenisolerades bara få patogener. De mest allmänna bakterieisolaten var Mycoplasma dispar och Pasteurellamultocida tillsammans. Alla Pasteurella sp. isolat var känsliga för testade antibiotika. Mannheimia haemolytica och M. bovis kunde inte påvisas i något prov.

## Introduction

Bovine respiratory disease (BRD) is a major health problem of cattle worldwide. It inflicts considerable financial losses in beef herds [15, 13] and is the most common cause of mortality in dairy cattle [35]. It is also an important welfare problem of calves. The causation is multifactorial and the disease appears to be a result of the interaction of infectious micro-organisms and such predisposing factors as host defence, environment and stress [22, 35]. Only a few reports exist on respiratory diseases of cattle in Finland [24, 28, 21]. In addition, limited field studies have been published from other Nordic countries [6, 30, 18, 10]. Finland has a special situation, with freedom from certain aetiological agents. Infectious bovine rhinotracheitis (IBR, BHV-1), for instance, does not exist in Finland. Moreover, *Mycoplasma* bovis has not been detected in Finnish cattle, and bovine virus diarrhoea (BVD) is very rare [28, 1]. Vaccines against respiratory disease are not used. Antimicrobials are generally not used for disease prevention and sick animals are mostly treated individually with antibiotics. However, the traditional farming system in Finland with small isolated cattle herds is changing. The dairy herds are enlarging gradually and calves of different ages are kept in group pens. In the new rearing system young calves at the age of 1–3 weeks originating from several dairy herds are transported to rearing units and reared in large groups. The aim of this study was to obtain basic knowledge of pathogens associated with bovine respiratory disease in Finland and to evaluate the occurrence of antimicrobial resistance in respiratory bacteria.

## Materials and methods

### Sample collection

This study was conducted from November 1998 to December 1999. Eighteen cattle herds situated in eastern, southern and western Finland were included. All herds had problems with bovine respiratory disease. Ten of the farms were rearing dairy-bred bull calves for beef, and 8 farms had dairy herds. The size of the beef-raising herds varied between 48 and 217 (mean 107) animals, and the dairy herds had 30–130 (mean 72) cows. Five diseased calves from each farm were chosen for closer examination, altogether 90 calves. A thorough clinical examination and tracheobronchial lavage were performed. The age of the diseased calves varied from 31 to 221 (mean 98) days and the weights were between 40 and 150 (mean 88) kg. All of the calves had abnormal sounds on auscultation of the respiratory tract, and most had either one or several of the following symptoms: fever >39.5°C, elevated respiratory rate (>40/min), cough or nasal discharge. One calf died 5 days after the examination and was autopsied. Blood samples for serological studies were taken from all calves at the beginning of the study, and second samples were taken from 86 calves 3–4 weeks later (paired serum samples). We also collected 6–10 blood samples from other animals of different ages on the farms to obtain more information about the serological situation of the whole herd. Some of the samples were taken from animals aged over 6 months to avoid the influence of maternal antibodies. The tracheobronchial lavage was taken with a special instrument made for collecting samples of the lower respiratory tract from calves [2]. The catheter was inserted nasally into the trachea. As lavage fluid, we used 30–40 ml of phosphate-buffered saline (PBS, Dulbecco's phosphate-buffered saline, Gibco TM, Invitrogen Corporation, Paisley, Scotland, UK). The tracheobronchial fluid was immediately aspirated through the catheter and removed into test tubes with a glucose calf serum (GS) broth for mycoplasma and transport media for bacteria isolation (Portagerm multi-transport medium BioMerieux, Lyon, France). The quantity of the collected fluid varied from 2 ml to over 20 ml.

### Detection of bacteria

Tracheobronchial lavage samples from 85 calves in 18 herds were tested for bacterial growth. Samples were cultured on blood agar (tryptose-soya-agar containing 5% bovine blood) aerobically and anaerobically as well as on selective agar for *Histophilus somni* (formerly *Haemophilus somnus*) in a CO_2_-enhanced atmosphere. More specific identification was made with biochemical tests. The susceptibility of different *Pasteurella* sp. isolates for selected antimicrobials was tested. The antimicrobials used were ampicillin, penicillin, trimethoprim-sulfamethoxazole, gentamicin, tetracycline and enrofloxacin or ciprofloxacin.

The bacterial tests were performed according to the standard procedures of the National Veterinary and Food Research Institute in Helsinki. The mycoplasma samples were kept frozen at -70°C until cultured. The media, culturing and identification methods used have been described elsewhere [27]. Species identification was based on growth characteristics and the selective use of an epi-immunofluoresence (IF) test; only antisera against *Mycoplasma dispar* 462/2, *Mycoplasma bovirhinis* PG 43 and *M. bovis* Donetta PG 45 were used.

### Detection of viral antibodies

Altogether 345 serum samples from 259 different animals were serologically tested for viral antibodies. Ninety samples were collected from diseased calves, 86 samples were paired serum samples from the same calves and 169 samples were taken from other cattle in the same herd. From each farm, 7–8 samples were from calves and 5–8 samples were from animals aged over 6 months. The serum samples were frozen at -20°C. They were tested for antibodies to bovine parainfluenza virus-3 (PIV-3), bovine respiratory syncytial virus (BRSV), bovine coronavirus (BCV), bovine adenovirus-3 (BAV-3) and bovine adenovirus-7 (BAV-7). In addition, 123 samples from 94 animals were tested for antibodies to bovine virus diarrhoea virus (BVDV). An ELISA test was used for antibodies to PIV-3, BRSV, BCV and BVDV. A virus neutralization test was used for BAV-3 and BAV-7. The ELISA kits (SVANOVA Biotech, Uppsala, Sweden) were used according to the manufacturer's instructions. If a fourfold increase occurred in antibody titre in the neutralization test or if the ELISA test was seronegative in the first sample and seropositive in the second sample, the calf was considered to have a rise in titre and to be recently infected with the relevant virus.

## Results

Results of the antibodies to viruses are presented in Table 1. Antibodies to PIV-3 and BAV-7 were found in all 18 herds. Antibodies to BCV and BAV-3 were discovered in 16/18 (89%) and from 15/18 (83%) herds, respectively. Antibodies to BRSV were found in 12/18 herds (67%) and in all dairy herds. Antibodies to BVDV were detected in 0/7 herds. High levels of antibodies to BAV-7 in more than 2 samples in one herd were monitored on 14 farms (neutralization titre more than 1:512). In paired serum samples, a rise in titres occured in 33 of 86 calves (33%) on 15 farms. Seroconversion for BAV-7, BAV-3, BCV and PIV-3 was seen on 10/18, 5/18, 3/18 and 2/18 farms, respectively. Titre rise for BAV-7 was most often seen on beef farms (8 herds) and titre rise for BAV-3 was most frequent on dairy farms (3 herds). Seroconversion to BRSV was not noted at any of the farms. Decreasing antibody titres (neutralization test) or a change from seropositive in the first sample to seronegative in the paired serum sample (ELISA test) was observed in 44 calves (51%) on 15 farms.

**Table 1:** Serological findings of 18 cattle herds with respiratory problems.

	No. calves (herds)	PIV3	BCV	BRSV	BAV3	BAV7
No. of herds positive	18	18	16	12	15	18
Percentage of positive herds	100	100	89	67	83	100
No. of diseased calves,^1^ first sampling	90 (18)	66 (18)	35 (15)	8 (6)	46 (13)	65 (17)
		73%^2^	38%	9%	51%	72%
No. of diseased calves,^1^ second sampling	86 (18)	58 (17)	30 (15)	5 (5)	44 (11)	72 (17)
		67%	35%	6%	51%	84%
No. of other animals^1^	169 (18)	150 (18)	57 (14)	18 (9)	107 (15)	143 (18)
		89%	34%	11%	63%	85%
Rise in titre^1^	86 (18)	3 (2)	4 (3)	0 (0)	11 (5)	20 (10)
		3.5%	4.7%	0%	12.8%	23.3%
Decrease in titre^1^	86 (18)	9 (7)	8 (6)	3 (2)	23 (10)	20 (8)
		10.5%	9.3%	3.5%	26.7%	23.3%

^1^ Number of herds in parentheses. ^2^ Percentage of calves

All 18 herds and 93% of the calves (79/85) had mycoplasma findings. *M. dispar* was found in 17 herds (94%) from 77 samples (91%), and other mycoplasmas in 16 herds (89%) from 52 samples (61%). On 15 farms (83%) and in 50 samples (59%), *M. dispar* occurred together with other mycoplasmas. All classical colonies examined by the IF test proved to be *M. bovirhinis. M. bovis* was not detected. Other bacterial growth was found in 9 herds (50%) in 18 samples (21% of calves). The most common finding was *Pasteurella* sp., which was monitored in 14 samples (78% of positive samples) of 8 herds (44% of the herds and 17% of the calves). The *Pasteurella* sp. isolates were identified as *P. multocida* in 7 cases and *P. multocida* (indole-negative) in 6 cases. Most often *Pasteurella* sp. was the only finding, but in 2 cases it was found together with *Fusobacterium necrophorum* and once with *Arcanobacterium pyogenes. F. necrophorum* alone was found from 3 samples in 3 herds and *A. pyogenes* alone only from one calf in one herd. All calves with other bacteria in tracheobronchial fluid also had mycoplasmal growth. *M. dispar* and *P. multocida* together were isolated in 13 samples from 7 herds. Six calves of these 13 showed a seroconversion to viruses, 4 of them to BAV-3, one to BAV-7 and one to BCV and BAV-7 simultaneously. The post-mortem examination of the dead calf revealed signs of chronic fibrinopurulent bronchopneumonia. *H. somni* was cultured from the lungs, in contrast to *P. multocida* (indole-negative), which was found earlier in the tracheobronchial lavage fluid. All 14 *Pasteurella* sp. strains were susceptible in vitro to all antimicrobials tested (ampicillin, penicillin, trimethoprim-sulfamethoxazole, gentamicin, tetracycline, enrofloxacin or ciprofloxacin).

## Discussion

According to the serological findings, PIV-3, BAV-7, BAV-3, BCV and even BRSV were common pathogens in Finnish cattle herds with respiratory problems. Finland is free from IBR and the occurrence of BVD is very rare [1]. Both of these viruses have been considered to be important BRD-associated pathogens elsewhere [35, 8, 20, 19]. Our negative findings confirm that BVDV infection is not closely linked to occurrence of BRD in Finland. The different serotypes of bovine adenovirus are divided into 2 subgroups. Types 1–3 represent group A and types 4–9 group B [14]. We chose to examine types 3 and 7. Our results agree with the findings of [24], who demonstrated that infections with BAV-1, -2 and -3 are common in cattle, at least in the southwestern part of Finland. To our knowledge the occurrence of BAV-7 has not been studied earlier in Finland. Here, seroconversion to BAV-7 was the most common, followed by seroconversion to BAV-3. Many of the older animals, especially on the beef farms, had high titres of BAV-7, which may indicate that an acute infection caused by this virus has occured. Antibodies to PIV-3 were found on every farm examined, and almost 80% of all samples taken were positive for PIV-3. This is in agreement with the ubiquitous nature of the virus, with its world-wide distribution [4, 8]. However, PIV-3 was not observed to be a causal factor. BCV has gained ground as a pathogen in respiratory disease complex recently [25, 11, 26]. BCV may also be involved in respiratory disease in Finnish cattle. Antibodies to BRSV were found in 67% of the herds overall and in all dairy herds. However, BRSV did not appear to cause the disease in the calves studied. The situation might have changed after the respiratory disease outbreak in Finland in spring 2000; the causative agents of the epidemic were suggested to include BRSV and BCV [21]. Although we detected antibodies to several viruses on each farm and even in high titres, we relatively seldom found seroconversion in the diseased calves. This is probably because of the young age of the calves and existing maternal antibodies which may suppress the calves' own production of antibodies. This is in agreement with the results of a high number of decreasing antibody titres and with the conclusion of [31, 32], who stated that young pneumonic calves often fail to sero-convert to agents present in respiratory tract because of suppressive maternal antibodies. Our failure to detect seroconversions might also be a consequence of missing the acute phase of the disease. Seroconversion to adenoviruses was seen in about half of the cases where also bacterial growth of *M. dispar* and *P. multocida* was noticed. There might have been some other predisposing factors than the viruses examined. Interpreting the importance of viral infection in this study is, however, problematic because we could not use any direct virus diagnostics from the tracheobronchial lavage and we did not have healthy calves or healthy herds as controls. Mycoplasmas are considered to be one of the pathogens causing BRD. They are able to cause a mild respiratory disease by themselves, but more often they are isolated from pneumonic lungs together with other pathogens [34, 12, 33]. The most common isolate from lavage fluid of diseased calves in our study was *M. dispar*. The predominant combination was *M. dispar* and *P. multocida*. In another study, a synergistic effect between *Mycoplasma* sp. and *P. multocida* was suggested [31]. We found no *M. bovis* which has not to date been isolated from Finnish cattle, and most likely it does not exist in Finland. However, many reports are available from other countries about the increasing prevalence of *M. bovis* [3, 9, 5]. The findings of *M. dispar* and *Mycoplasma* sp. did not differ much from previous reports in Finland. [28] described a high prevalence of mycoplasma from nasal and transtracheal samples, and rapid spread of the infection in a population of young cattle. In our study, other bacteria were detected in only 21% of the tracheobronchial samples. [31] found in transtracheal wash samples (TTW) bacterial growth in 90% of the diseased calves and in 55% of the controls. *M. haemolytica* biotype A serotype 1 is considered to be the predominant and most pathogenic bacterium in pasteurellosis of feedlot units [7, 16]. In our samples, we found no *M. haemolytica*.

*P. multocida* is another common pathogen reported to be involved in BRD, especially in dairy herds [31, 16]. Some of our *Pasteurella* sp. strains differed in biochemical tests from the typical *P. multocida* by showing a negative reaction to indole. An important finding was that all Pasteurella isolates were susceptible to all of the antimicrobials typically used in the treatment of BRD in Finland. An explanation for the very few bacterial findings in spite of mycoplasmas may lie in the lavage technique and the relatively small fluid volume used. Alternatively, perhaps few pathogens were present in the tracheobronchial area at the time of the investigation. The postmortem finding of *H. somni* may indicate that we failed to detect some bacteria in the tracheobronchial area. However, the calf may have contracted the *H. somni* infection after the lavage and the *P. multocida* infection. [29] described *H. somni* as a frequent finding in autopsied calves with respiratory disease in Denmark. *H. somni* has seldom been found in Finland, with only 10 reports in the last 10 years and only in connection with respiratory disease [17]. Isolation of *A. pyogenes* and *F. necrophorum* without any other bacteria was considered to be a contamination or a coincidental finding. No connection to the poor prognosis of calves with *A. pyogenes* was seen, in contrast to [23] and [16]. The scant bacterial findings and low mortality in our study may also indicate that the severity of the disease was lower than in many other studies.

## Conclusion

According to serological findings, BAV-7, PIV-3, BAV-3, BCV and BRSV are common viruses in Finnish cattle herds suffering from respiratory disease. Seroconversion to BAV-7 and BAV-3 occurred most often. These results suggest an active role of adenoviruses, particularly BAV-7, in respiratory diseases in cattle in Finland. Antibodies to BRSV were detected mainly in older animals without signs of respiratory disease and no seroconversion. Tracheobronchial lavage revealed few pathogens other than mycoplasmas, which could be found in all herds in this study. The most common bacterial findings were *P. multocida* and *M. dispar*, suggesting their importance in respiratory problems. How these 2 pathogens interact together and with viral pathogens remains to be clarified. *M. haemolytica* and *M. bovis* were not found, and *H. somni* was only found in the lungs of the autopsied calf. New calf-rearing systems, where large groups of young calves are reared in close proximity, may increase respiratory problems considerably also in Finland. Larger studies which will enable more information to be gathered about respiratory diseases and the roles of different pathogens are therefore warranted.
